# Periodic Nucleation of Calcium Phosphate in a Stirred Biocatalytic Reaction

**DOI:** 10.1002/anie.201911213

**Published:** 2020-01-09

**Authors:** Bíborka Bohner, Tamás Bánsági, Ágota Tóth, Dezső Horváth, Annette F. Taylor

**Affiliations:** ^1^ Department of Physical Chemistry and Materials Science University of Szeged Rerrich Béla tér 1. 6720 Szeged Hungary; ^2^ Department of Applied and Environmental Chemistry University of Szeged Rerrich Béla tér 1. 6720 Szeged Hungary; ^3^ School of Chemistry University of Birmingham Edgbaston Birmingham B15 2TT UK; ^4^ Department of Chemical and Biological Engineering University of Sheffield Mappin Street Sheffield S1 3JD UK

**Keywords:** biocatalysis, biomineralization, reaction networks, self-organization, systems chemistry

## Abstract

Highly ordered superstructures composed of inorganic nanoparticles appear in natural and synthetic systems, however the mechanisms of non‐equilibrium self‐organization that may be involved are still poorly understood. Herein, we performed a kinetic investigation of the precipitation of calcium phosphate using a process widely found in microorganisms: the hydrolysis of urea by enzyme urease. With high initial ratio of calcium ion to phosphate, periodic precipitation was obtained accompanied by pH oscillations in a well‐stirred, closed reactor. We propose that an internal pH‐regulated change in the concentration of phosphate ion is the driving force for periodicity. A simple model involving the biocatalytic reaction network coupled with burst nucleation of nanoparticles above a critical supersaturation reproduced key features of the experiments. These findings may provide insight to the self‐organization of nanoparticles in biomineralization and improve design strategies of biomaterials for medical applications.

## Graphical abstract

**Periodic precipitation** of calcium phosphate was obtained by using a biocatalytic reaction to maintain the system far from equilibrium. A simple model involving burst nucleation of nanoparticles above a critical supersaturation reproduced the results, suggesting a potential mechanism for self‐organization in biomineralization.

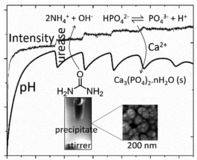

## Introduction

Self‐organization refers to the structures found in chemical and biological systems arising from internal feedback mechanisms far from equilibrium.^1^ Some interesting examples of self‐organization in materials have been obtained, inspired by natural processes such as the oscillatory regulation of microtubule growth in cells or the formation of coccoliths in marine algae.^2^ Understanding how self‐organization arises in nature will aid in the design of bioinspired materials for widespread applications.^3^

Many natural and synthetic processes involving mineral precipitation develop remarkable superstructures composed of nanoparticles.^4^, ^5^ Complex shapes, known as biomorphs, form when barium carbonate nanocrystals precipitate in silica solution.^6^ Periodicity has been observed in the arrangement of calcite nanocrystals in calcium carbonate composites^7^ and in chemical garden structures^8^ or Liesegang patterns^9^ driven by the coupling of chemical and physical processes. In these synthetic systems, mass transport plays an important role in the development of structure. Herein we report the first observations of periodic precipitation in a stirred biocatalytic reaction. The results illustrate how coupled chemical reactions can be used to generate oscillations in the production of nanoparticles, driven by competition between a reaction producing inorganic ions and a phase change process consuming them.

Bacteria use networks of enzyme‐catalyzed reactions to drive mineral formation. One well known reaction is that of urease which breaks down urea and forms ammonia, raising the pH.^10^ This leads to precipitation resulting in stone formation and catheter blockages. Urease has also been used to produce calcium carbonate and other minerals including calcium phosphate, both as a study of the biomineralization process but also for applications in engineering such as cement strengthening.^11^

We investigated the kinetics of reaction‐induced precipitation of calcium phosphate, driven by urease. Calcium phosphate is an important biomineral produced by bacteria, often found intracellularly as amorphous calcium phosphate (ACP), a precursor to the crystalline forms.^12^ ACP is also implicated in the early stages of bone development in vertebrates.^13^ There are numerous methods for the synthesis of calcium phosphate and its various polymorphs in the laboratory, but fewer studies modelling its formation, particularly under biologically relevant conditions.^14^ It is widely used in biomaterials applications such as bone and dental cements and coatings for metallic prostheses.^12^

We found that under certain conditions periodic precipitation of calcium phosphate occurred along with pH oscillations. Internal pH changes caused by competing reactions—production of ammonia and removal of phosphate—resulted in periodic changes in supersaturation and nucleation of nanoparticles. The results were reproduced in a simple model inspired by the LaMer theory for the formation monodisperse colloids involving burst nucleation above a critical supersaturation.^15^ Our results may help uncover the mechanisms that give rise to self‐organization in some natural and synthetic biomineral systems.

## Results and Discussion

Precipitation of calcium phosphate was achieved by addition of a solution of the enzyme urease (Sigma‐Aldrich, Type III) containing sodium phosphate to a stirred solution of urea, calcium chloride and hydrochloric acid (see Experimental Section and Supporting Information). For certain initial concentrations, there was a delay before the increase in pH associated with the enzyme catalyzed hydrolysis of urea:

(1) $$\mathrm{CO}\left(\mathrm{NH}{}_{2}\right){}_{2}\;+\;H{}_{2}O\;\overset{\mathrm{urease}}{\underset{}{\to }}\;2\;\mathrm{NH}{}_{3}\;+\;\mathrm{CO}{}_{2}$$

**Figure 1:**
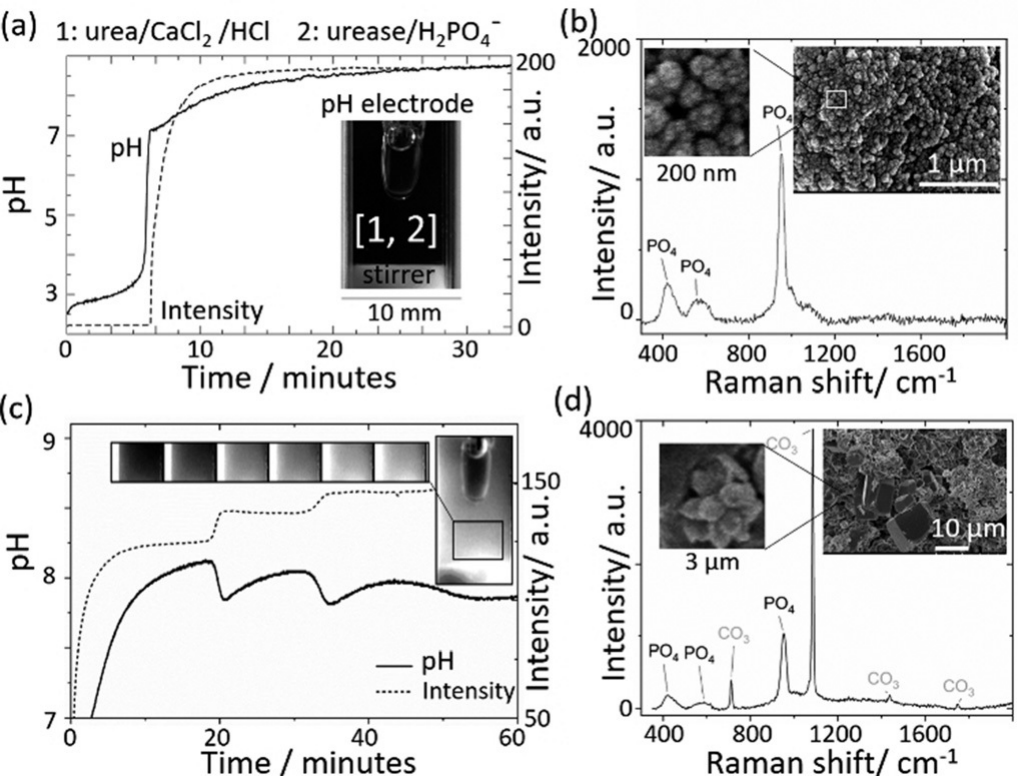
Precipitation of calcium phosphate in a cuvette with high stirring rate (1000 rpm). a) Changes in pH and intensity of grayscale images with initial concentrations of [urea]=0.08 m, [CaCl_2_]=4 mm, [HCl]=3 mm, [urease]=20 u mL^−1^, [H_2_PO_4_^−^]=0.034 M. b) Raman spectrum with PO_4_ peaks at ≈420, 586 and 952 cm^−1^ and SEM of precipitates formed with concentrations in (a). c) Periodic, stepwise precipitation and pH oscillations with [urea]=0.5 m, [CaCl_2_]=0.25 m, [HCl]=5 mm, [urease]=20 u mL^−1^, [H_2_PO_4_^−^]=0.034 M. d) Raman spectrum from precipitate taken during the reaction with concentrations in d) except [urease]=30 u mL^−1^ and [H_2_PO_4_^−^]=0.05 m with broad PO_4_ peaks at ≈420, 586 and 952 cm^−1^ and CO_3_ peaks at 712, 1086, 1437 and 1747 cm^−1^. Inset: SEM of precipitates.

Rapid precipitation was observed when the pH reached 7, and there was a corresponding change in the intensity of images of the cuvette (Figure 1 a). Turbidity measurements revealed an increase in the amount of calcium phosphate formed with higher initial concentrations of calcium ion (Figure S1). The washed and dried particles were <100 nm in diameter with a spherical morphology (SEM in Figure 1 b and Figure S2). The bands obtained in the Raman spectrum of the particles are associated with vibrational modes of PO_4_^3−^; the broad peak at 952 cm^−1^ (Figure S3) is indicative of amorphous calcium phosphate (ACP) or poorly crystalline hydroxyapatite (Ca_10_(PO_4_)_6_(OH)_2_, HAP).^16^ Biological macromolecules and carbonates can inhibit crystal growth so the enzyme urease or the carbonate produced from hydrolysis of urea may aid in the stabilization of ACP.^17^

When the concentrations of urea and calcium chloride were increased, a stepwise production of precipitate was observed (Figure 1 c), accompanied by oscillations in pH between 7.8 and 8.1. The Raman spectra of the dried precipitate taken during the oscillatory reaction contained a phosphate band at 952 cm^−1^ and characteristic calcite peak at 1086 cm^−1^, suggesting a mixture of amorphous calcium phosphate and calcite was formed (Figure 1 d and Figure S4). The SEM of the dried precipitates showed submicron particles and micron sized rhombic structures (Figure 1 d and Figure S5).

The range of concentrations for which oscillations were observed is shown in Table 1 and typical pH/intensity plots are included in Figure S6–S8. In all cases, precipitate was obtained when the pH increased above 7, but stepwise production commenced with a sharp drop in the pH when the pH reached ≈8 to 8.4. The average amplitude of the pH oscillations was 0.3±0.08 and did not vary greatly between experiments, but dampened as the reaction progressed. The period of oscillations was between 5 to 20 minutes and in general was higher with decreases in [Ca^2+^] or [urea]. The period and number of oscillations were found to be particularly sensitive to the nature of the stirring, including the type of magnetic stirrer bar used and the stirring rate.

**Table 1:** Range of concentrations where oscillations were obtained under well‐stirred conditions (1000 rpm). HCl=5 mm in all cases.

[urease] [u mL^−1^]	[H_2_PO_4_^−^] [m]	[urea] [m]	[CaCl_2_] [m]	⟨Period⟩ [min]
10–40^[a]^	0.017–0.07	0.5	0.25	7–18
30^[b]^	0.052	0.5	0.125–0.75	7–20
20^[c]^	0.034	0.5–1	0.25	5–13

[a] Figure S6. [b] Figure S7. [c] Figure S8 for typical plots.

**Figure 2:**
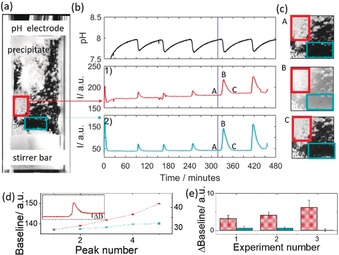
Periodic nucleation and aggregation of calcium phosphate in a cuvette with low stirring rate (200 rpm) where [urea]=0.5 m, [HCl]=5 mm, [CaCl_2_]=0.25 m, [urease]=30 u mL^−1^, [H_2_PO_4_^−^]=0.05 M. a) image of the cuvette where the white agglomerates of precipitate are deposited on the walls and pH electrode. b) Time series of pH, 1) average intensity of images of wall‐coating aggregates in the red box and 2) average intensity in images of the bulk solution in the blue box. The blue line indicates the time point at which the image on the left was taken. c) images of part of the cuvette at time points indicated A, B and C. d) Baseline intensity after a peak from an area in the cuvette with aggregate (red) and without aggregate (blue) for experiment in (a). Inset shows peak and change in baseline intensity. e) Average change in baseline intensity from areas with aggregate (red) and areas without aggregate (blue) for three repeats of the experiment. Error bars indicate standard deviation calculated from multiple sample areas.

If the stirring rate was reduced such that aggregation could occur, clusters of particles deposited on the electrode and walls of the cuvette (Figure 2). The amplitude of pH oscillations was the same as with higher stirring rate and the period of oscillations was greater. We found that there was a total increase in the amount of precipitate in time, indicated by the stepwise change in baseline intensity (red box around wall‐coating aggregates in image and red intensity curve 1 and Figure 2 d). However, in the bulk solution oscillations in intensity were observed with a return to the same baseline intensity and an absence of precipitates (blue box around bulk solution in image and blue intensity curve 2). In repeat experiments, there was a change in baseline intensity after a peak in areas of the cuvette with aggregate, whereas in areas without aggregate it was close to zero (Figure 2 e and Figure S9). This suggests that the periodicity arose from rapid nucleation of calcium phosphate in the bulk, stirred solution.

In order to gain a better understanding of the processes involved, we devised a simple model of the reaction. The model included the hydrolysis of urea by urease with well‐known kinetics and the ammonia, carbonate and phosphate equilibria (see supplementary information, section 3). In addition, we included precipitation of calcium phosphate and calcium carbonate. We note that it is likely there are multiple calcium phosphate species formed and there are various types of ACP including amorphous tricalcium phosphate (ATCP):^18^

(2) $$3\;\mathrm{Ca}{}^{2+}\;+\;2\;\mathrm{PO}{}_{4}{}^{3-}\;+\;n\;H{}_{2}O\;\to \;\mathrm{Ca}{}_{3}\left(\mathrm{PO}{}_{4}\right){}_{2}\cdot n\;H{}_{2}O\;\left(s\right)$$

where some PO_4_^3−^ ions can be replaced by HPO_4_^2−^ and OH^−^ or other ions giving different ratios of [Ca]/[P]_T_ and [P]_T_ is the total concentration of phosphorous.

The solubility product *K*_sp_ for amorphous compounds cannot be precisely determined but a range of estimates have been reported, from 3×10^−17^ to 2×10^−33^.^19^ For simplicity, we included only ATCP in the model and took the value of *K*_sp_=3×10^−17^. Calcium carbonate also has various polymorphs; here there was evidence of calcite from the Raman and SEM, hence we used the *K*_sp_ corresponding to that of calcite (*K*_sp_=3.3×10^−9^), although this may also be preceded by less stable amorphous species.^20^

The rate of formation of solid depends on the supersaturation, *S*, defined here as the ratio of the ion product to the solubility product:

(3) $$S=IP/K{}_{\mathrm{sp}}\;\;IP=\left\{\mathrm{Ca}{}^{2+}\right\}{}^{3}\left\{\mathrm{PO}{}_{4}{}^{3-}\right\}{}^{2}$$

where {} denotes activities of the ions in solution. Precipitation is thermodynamically favored for *S*>1 and typically involves primary and secondary nucleation, growth, aggregation/breakage and Ostwald ripening. We consider only primary nucleation here as the experiments with slow stirring rate suggest that this is the driving force for periodicity. To imitate burst nucleation, the rate of production of spheres of precipitate of a critical radius, *r*_c_, was approximated by:

(4) $$\frac{d\left[ATCP\right]}{dt}=p\left(S\right)K_{J}S\;exp\left(-\frac{B}{{\left(lnS\right)}^{2}}\right)$$

where *K*_J_=*AV*_c_/*V*_m_, and *V*_c_ is the volume of a particle of radius *r*_c_ and *V*_m_ is the molar volume. The exponential term and parameters *A* and *B* arise from classical nucleation theory. The probability term, *p*(*S*), was introduced in order to take into account the onset of nucleation at a critical value of *S*:

(5) $$S\;\geq \;S{}_{\mathrm{crit}1}\;\;p=1$$

where the value of *S*_crit1_ was given by *S*_crit1_=1+ *R*_1_[Ca^2+^]/[PO_4_^3−^] and *R*_1_ is a constant. The nucleation was terminated when *S* ≤ *S*_crit2_ (given by 1 + *R*_2_[Ca^2+^]/[PO_4_^3−^], where *R*_2_ ≪ *R*_1_)_._ The dependence of *S*_crit_ on the ratio [Ca^2+^]/[PO_4_^3−^] was intended to take into account the fact that probability of precipitation was more likely as the concentration of [PO_4_^3−^] was increased for [Ca^2+^] > [PO_4_^3−^] that is, *S*_crit_ decreased.^21^ Further details of the model and all parameters are given in the supplementary information.

**Figure 3:**
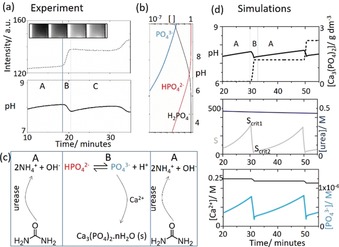
Periodic precipitation split into time zones A and B and a) intensity and pH from the experiments in Figure 1 c. b) Distribution of phosphate species as a function of pH calculated from the various equilibria, c) basic mechanism of pH increase in A and pH decrease in B, d) simulations showing pH and [ATCP] in time, urea and supersaturation S, and calcium and phosphate for initial concentrations: [urea]=0.5 m, [HCl]=5 mm, [CaCl_2_]=0.25 m, [urease]=30 u mL^−1^, [H_2_PO_4_^−^]=0.05 m.

The oscillatory process observed in experiments can be split into two zones, A and B, as shown in Figure 3 a. In zone A, there was no nucleation, and the pH was increasing as a result of the urease reaction (reaction 1) and the production of ammonia. In zone B, there was fast precipitation of calcium phosphate and the pH dropped. The precipitation then terminated, there was no more nucleation and the pH again increased. The distribution of phosphate species is shown in Figure 3 b and the key processes in the model are in Figure 3 c. The concentration of PO_4_^3−^ increased when the pH reached high values. Then removal of PO_4_^3−^ by reaction with Ca^2+^ resulted in a pH drop, that is, the net process in B could be written as:

(6) $$3\;\mathrm{Ca}{}^{2+}\;+\;2\;\mathrm{HPO}{}_{4}{}^{2-}\;+\;n\;H{}_{2}O\;\to \;\mathrm{Ca}{}_{3}\left(\mathrm{PO}{}_{4}\right){}_{2}\cdot n\;H{}_{2}O\;\left(s\right)\;+\;2\;H{}^{+}$$

with the rate‐determining nucleation reaction involving PO_4_^3−^. The model was able to reproduce oscillations in pH and a stepwise production of precipitate (Figure 3 di). The simulations suggested that only a small decrease in urea was observed during the oscillatory reaction (Figure 3 dii). The supersaturation, *S*, increased as a result of the greater concentration of PO_4_^3−^ at higher pH. Nucleation was suppressed until the critical value S_crit1_. At this point, the probability of nucleation was 1 and a rapid drop in *S* and pH was observed, along with consumption of PO_4_^3−^ (Figure 3 diii). When *S* fell below *S*_crit2_, nucleation terminated. The enzyme reaction then dominated again, with a renewed rise in pH. Hence the instability occurs as a result of two competing processes that cause PO_4_^3−^ formation and depletion.

**Figure 4:**
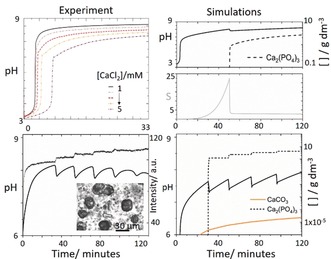
Comparison of reaction profiles in experiments and simulations for different reaction conditions a) pH profile in experiments with low calcium chloride concentration where [urease]=20 u mL^−1^, [H_2_PO_4_^−^]=0.034 m, [urea]=0.08 m, [HCl]=3 mm, and [CaCl_2_]= 1–5 mm, b) simulations with same conditions as in Figure 3 d but reduced calcium chloride [CaCl_2_]=0.03 m. c) oscillations with [urea]= 0.5 m, [CaCl_2_]=0.25 m, [HCl]=5 mm, [urease]=30 u mL^−1^, [H_2_PO_4_^−^]=0.05 m. Inset shows optical microscope image of a sample of the reaction after oscillations with small scale precipitates (calcium phosphate) and larger structures (calcite) (d) Simulations showing production of calcium phosphate (dashed) and calcite (orange) with [urea]=0.5 m, [CaCl_2_]=0.25 m, [HCl]=5 mm, [urease]=30 u mL^−1^, [H_2_PO_4_^−^]=0.05 m.

In order for oscillations to arise, the rate removal of phosphate must overcome the rate of production of phosphate from the biocatalytic process. When the ratio of [Ca]/[P]_T_ was reduced, nucleation occurred at lower values of *S*_crit1_ and the rate was reduced such that *S* fell to a steady state value above *S*_crit2_. Continuous rather than periodic nucleation was observed, in agreement with experiments (Figure 4 a,b). If the enzyme rate (turnover number) was increased in simulations, or the precipitation rate (*K*_J_) decreased, then *S* rose above *S*_crit1_ and continuous production of calcium phosphate was also observed. Hence oscillations only occurred for a finite range of values of *S*_crit1_ that depended on the composition of the reaction mixture. We found that in simulations the amplitude of pH oscillations was governed primarily by *S*_crit1_, and hence the ratio of [Ca]/[P]_T_ and the absolute pH by *K*_sp_ and the enzymatic rate. The period was governed by a combination of the enzyme rate and the nucleation rate, both of which are sensitive to the stirring rate.

Calcium carbonate was formed after calcium phosphate as revealed by SEM (Figure S10), and analysis of a sample taken after the oscillations with an optical microscope revealed that growth of crystals of CaCO_3_ occurred at later stages of the reaction, when nucleation and growth of ATCP appeared to have terminated. When calcium carbonate nucleation was included in the model, this species was produced more gradually than ATCP (Figure 4 d) and did not form stepwise—hence it is unlikely that it contributed to the observed periodicity. The pre‐exponential *A* factor is expected to be smaller for a crystalline form than for an amorphous phase, and the rate of nucleation of calcite was much smaller than that of ATCP. This meant that the supersaturation was always above the threshold for calcite, and continuous nucleation was observed.

Transformations between phosphate polymorphs are known to display autocatalytic features: a sigmoidal change in [Ca^2+^], and enhanced rate in the presence of the crystalline product.16a, 22 These processes likely occur by internal rearrangement and expulsion of acid. Oscillations have not previously been reported, probably because such systems either do not involve continuous reactive supply of ions or employ the relatively high metal:counterion ratio used here.11b In addition, the burst nucleation observed here occurred on a faster timescale.

In classical nucleation theory, the steady state nucleation rate is calculated from the number of clusters above a critical radius. Both the critical nucleus size and the induction period before the observable appearance of precipitate decrease with increasing *S*. However, we found that the inclusion of a critical supersaturation, *S*_crit_, for nucleation was essential in the model in order to reproduce the oscillations. LaMer proposed that for systems in which the solute is supplied by for example, chemical reaction, the increase in *S* takes the system into a metastable region where precipitation is negligible until a critical supersaturation, *S*_crit_, is reached and then rapid nucleation occurs.15a This removes ions from solution until *S* falls below *S*_crit_ where nucleation is terminated and growth of crystals can then occur. The LaMer model separates the processes of nucleation and growth and is often used to describe nanoparticle synthesis, where burst nucleation results in uniform size particles from a near homogeneous solution.15b We have used a similar approach for modelling nucleation of nanoparticles of ATCP. The probability function used here allowed for a dynamic *S*_crit_ that depended on the solution composition.

The time before *S*_crit_ is reached may involve the establishment of stable pre‐nucleation clusters as the formation of ACP is believed to occur via Posner clusters—Ca_9(_PO_4_)_6_—of size 1 nm.^13^ Aggregates of various Ca:P stoichiometries have been reported in systems with different initial compositions and methods of preparation; and the presence of pre‐nucleation clusters was confirmed in these cases.19a Privman et al. proposed a kinetic model for aggregation via pre‐nucleation clusters in colloidal systems which resulted in a similar expression to the one used here.^23^ This expression did not give oscillations in our model without the inclusion of *S*_crit_. A much steeper dependence of the nucleation rate on *S* (e.g. *S* ^20^) has also been used to simulate the more step‐like dependence of nucleation rate on supersaturation in spatially distributed systems, as well as a Heaviside function (for *S* > *S*_crit_).^24^ This scenario appears relevant for solutions in which one ion is in large excess, and supersaturation is rising as a result of the increase in the counter‐ion, either by reaction or by diffusion.

Other precipitation models include autocatalysis in growth of colloids (Finke–Watzky model) and autocatalysis through secondary nucleation which is responsible for rapid precipitation in some inorganic salt systems.^25^, ^26^ However, the latter cannot explain the periodicity described here as the product particles were always present after the first nucleation event: there would be no time delay necessary for oscillations. Oscillations in precipitation and in nanoparticle aggregation have been obtained in stirred systems involving pH oscillators—in this case the driving force for the behavior was pH autocatalysis driven by inorganic chemistry in open flow reactors.^27^ Oscillations in pH have also been achieved with enzyme catalyzed reactions involving feedback through the rate–pH curve.^28^ Here we presented the first example of a system where the internally pH‐regulated formation of the counter‐ion, phosphate, along with burst nucleation are the driving force for the periodicity.

In unstirred systems, periodic precipitation is frequently obtained. Liesegang patterns have been modelled using the Cahn–Hilliard equation to effectively describe the spatial phase separation process.^29^ Biomorphs also show periodic nanoparticle deposition and local pH oscillations have been observed in experiments.^30^ The main difference between our system and these precipitation patterns is that spatial gradients and diffusion are responsible for the changes in supersaturation whereas here it was driven by chemical reaction. The role of the biocatalytic process, then, was to maintain the system far from equilibrium with the slow consumption of the chemical fuel, urea, that regulated the pH and hence the precipitation process under closed conditions.

## Conclusion

Here, we have obtained periodic nucleation of calcium phosphate and oscillations in pH by using a biocatalytic reaction to maintain the system far from equilibrium. We presented a simple model that reproduced the results by coupling the biocatalytic reaction to burst nucleation of nanoparticles above a threshold supersaturation, inspired by models for the formation of monodisperse colloids proposed by LaMer.

The mechanisms underlying oscillations in nanoparticle production described here may find more general applications. Coupled reaction networks, where the output of one process regulates the input of another, are frequently found in nature and self‐organization may be used to arrange supramolecular chemical structures such as nanoparticles into hierarchical structures. Transport processes such as diffusion will then play an important role. There are many natural materials that that involve periodic deposition of nanoparticles. It may be that the approach presented here can help describe some of these, or allow us to develop a strategy for the control of superstructures formed in synthetic, bioinspired mineral systems.

## Experimental Section

Two solutions were prepared in ultrapure water (Milli‐Q). In solution A, urea (Sigma–Aldrich), calcium chloride dihydrate (Sigma–Aldrich) and hydrochloric acid (Sigma–Aldrich) were dissolved. Solution B contained urease/phosphate (from *Canavalia ensiformis* (Jack bean) type III, Sigma–Aldrich, 31 430 u g^−1^, containing phosphate 0.17 mg/u). For experiments in the cuvette, one mL of B was added to one mL of A under constant stirring using a magnetic stirrer (HI‐190M) with a Spinfin® stirrer bar. Experiments were performed at room temperature (20±2 °C). The pH was monitored using a pH microelectrode (Mettler Toledo and Pico Data logger software) and images were taken using a PixeLINK® camera and analyzed using ImageJ or MATLAB. Turbidity measurements were obtained using a VWR UV‐3100PC spectrophotometer. The precipitate was filtered, rinsed with doubly deionized water and air dried. Raman spectroscopy was performed on a Thermo ScientificTM DXR^TM^ Raman microscope using a green laser (*λ*=532 nm), at 5 mW. Scanning electron microscopic (SEM) images were recorded by a Hitachi S‐4700 field emission scanning electron microscope, at 10 kV. Optical microscope images were produced from an aqueous sample of solution collected after oscillations ceased using a Leica TCS SP8 Confocal Microscope. For more details, see the supplementary information.

## Conflict of interest

The authors declare no conflict of interest.

## Supporting information

As a service to our authors and readers, this journal provides supporting information supplied by the authors. Such materials are peer reviewed and may be re‐organized for online delivery, but are not copy‐edited or typeset. Technical support issues arising from supporting information (other than missing files) should be addressed to the authors.

Supplementary
