# Supplementary material for: Periodic Nucleation of Calcium Phosphate in a Stirred Biocatalytic Reaction
Source: Angew Chem Int Ed Engl. 2020 Jan 9;59(7):2823–8. doi: 10.1002/anie.201911213 (PMC7027757; doi:10.1002/anie.201911213)
Supplement: Supplementary file 1 — Supplementary [file ANIE-59-2823-s001.pdf]

Angewandte Chemie
Eine Zeitschrift der Gesellschaft Deutscher Chemiker

Supporting Information

Periodic Nucleation of Calcium Phosphate in a Stirred Biocatalytic Reaction

*Bíborka Bohner, Tamás Bánsági, Jr., Ágota Tóth, Dezső Horváth, and Annette F. Taylor**

anie_201911213_sm_miscellaneous_information.pdf

Supplementary information

1. **Materials and methods**

Reactants were prepared in ultrapure water (Milli-Q); a stock solution A of calcium chloride dihydrate (Sigma Aldrich, meets USP testing specifications); hydrochloric acid (Molar, analytical reagent); and urea (Sigma Aldrich, meets USP testing specifications) and a stock solution B of urease enzyme (Sigma Aldrich, U1500, Type III powder, from *Canavalia ensiformis* (Jack Bean), 34310 u/g) which contains orthophosphate PO_4^{3-} (0.17 mg/unit, phosphate test kit Quantofix®). With the typical activity of 34310 units/g and M_r = 545 kDa = 545 x 10^3 g/mol for Jack Bean urease, an activity of 19 units/ml gives a maximum theoretical concentration (not taking into account the impurities) of enzyme = 0.545 g/L = 1 µM.

The reaction was performed in two configurations: a quartz cuvette (2 ml) or a 20 ml container which was either sealed (no air interface) or open to the atmosphere. Experiments were performed at room temperature of 20 ± 2 ˚C. In the quartz cuvette, one ml of solution B was added to one ml of solution A with constant stirring. The reaction was stirred using a HI-190M-0 1L Magnetic Mini Stirrer with Speedsafe and a Spinfin® magnetic stirring bar of diameter 10 mm. The stirring rate was either 1000 rpm or 200 rpm, with the cross-bars facing down in the latter to allow the aggregate to settle. We found that the nature of stirring and the presence of an open air interface had an effect on the induction time and period and number of oscillations but not the qualitative behaviour. These factors influence the biocatalytic reaction as there may be transfer of ammonia and carbon dioxide to the air, changing the solution pH which in turn affects the enzyme rate. The stirring rate is also expected to affect the secondary nucleation rate, as well as aggregation of the particles.

The progress of the reaction was followed with a pH microelectrode (Mettler-Toledo and Pico Data logger) with measurements taken every second. We obtained images every 10 s of the precipitation process in the reactor using a digital camera (PixeLINK®) and associated software. The RGB images were converted to greyscale and the average intensity of a selected area was extracted as a function of time using either ImageJ or MATLAB. An increase of intensity corresponded to the appearance of white precipitate. Six repeats were performed for the experiment in which aggregate was allowed to accumulate on the walls of the cuvette. The average intensity was determined from image areas with aggregate and compared to image areas without aggregate (Figure S9). The average baseline intensity, change in baseline and the standard deviation was extracted from three or more sample areas. We also carried out turbidity measurements (in 2 mL volume, at λ = 400 nm, and T = 25°C) in a

UV-vis spectrophotometer (VWR, UV3100-PC) in experiments with lower amounts of calcium ion where the absorbance is expected to be proportional to the amount of precipitate (Figure S1).

The precipitate was filtered and rinsed with doubly deionized water. After air drying, the powder was dispersed on carbon conductive adhesive tape, and coated with platinum and gold alloy. Raman spectroscopic studies were performed on a Thermo Scientific™ DXR™ Raman microscope using a green laser (λ = 532 nm), operating at 5 mW laser power. For each sample, 30 spectra were averaged with an exposure time of two seconds. Scanning electron microscopic (SEM) images were recorded by a Hitachi S-4700 field emission scanning electron microscope, operating at 10 kV acceleration voltage. Brightfield optical microscopy images were obtained using a Leica TCS SP8 Confocal Microscope.

The SEM of samples collected with low urea and low [Ca^{2+}] indicate formation of roughly spherical particles or aggregates less than 100 nm in size (Figure S2). The precipitate formed did not react with HCl and the Raman spectra indicated that it contained entirely calcium phosphate; there was no evidence of calcite under these conditions. There were typical peaks associated with PO_4 vibrational modes at 420 cm^{-1} and 580 cm^{-1}; the broad peak at 952 cm^{-1} (Figure S3) corresponds to the symmetric stretching mode (v_1) of the tetrahedral PO_4 group and is indicative of amorphous calcium phosphate (ACP) or poorly crystalline hydroxyapatite ($Ca_{10}(PO_4)_6(OH)_2$, HAP).[1] The absence of signals at 1103 cm^{-1} and 1073 cm^{-1} suggests there is no incorporation of carbonate ions in the samples.[2]

With higher [Ca^{2+}] and [urea], the precipitate reacted with HCl and the Raman spectra (Figure S4) showed that it contained a mixture of calcium phosphate with PO_4 peaks at 420, 580 and 952 cm^{-1} and calcium carbonate with CO_3 peaks at 712, 1086, 1437 and 1747 cm^{-1}. The characteristic strong peak at 1086 cm^{-1} is indicative of calcite. The SEM contained a mixture of submicron particles and micron scale crystals (Figure S5).

2. Experimental data

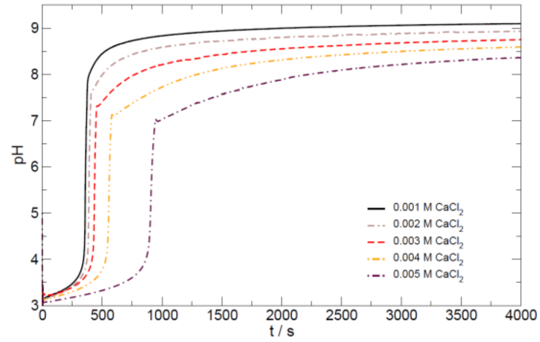

(a)

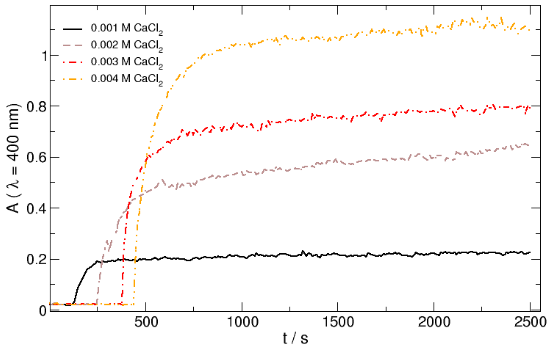

(b)

Figure S1. (a) Effect of increasing calcium ion concentration on the urea–urease reaction and (b) turbidity measurements at T = 25°C. The concentrations were [urease] = 20 u/ml, [$H_2PO_4^-$] = 0.034 M, [urea] = 0.08 M, [HCl] = 3 mM, and [$CaCl_2$] = 1 – 5 mM.

(a)

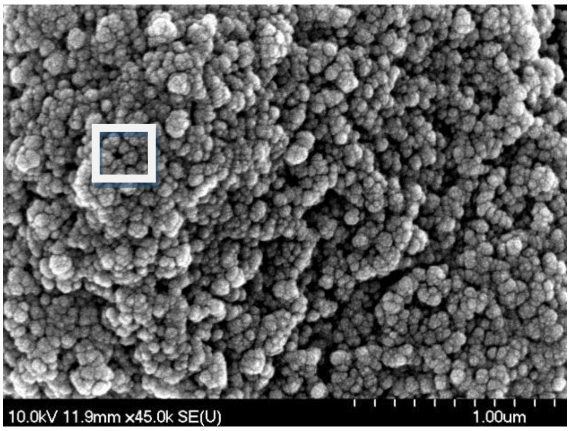

(b)

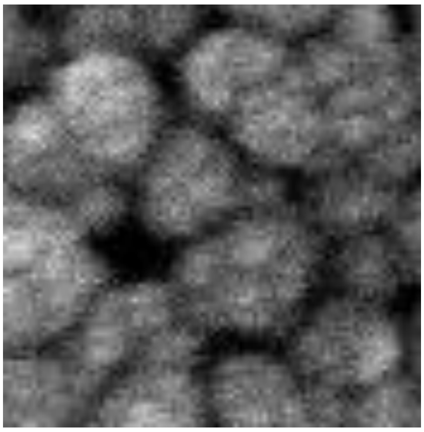

Figure S2. (a) SEM images of the precipitate formed in the reaction with [urease] = 20 u/ml, [$H_2PO_4^-$] = 0.034 M, [urea] = 0.08 M, [HCl] = 2.2 mM, and [$CaCl_2$] = 4 mM and (b) 200 nm x 200 nm section corresponding to the white square shown in (a).

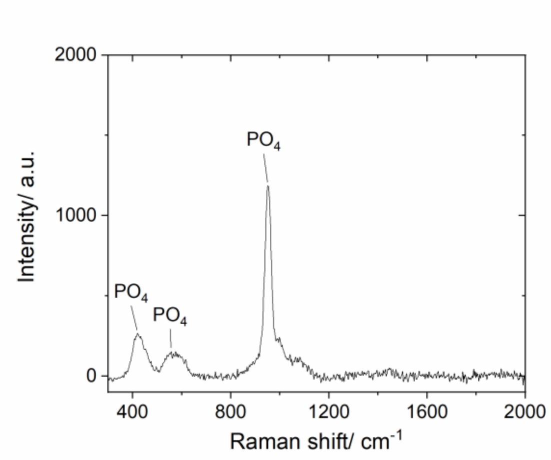

(a)

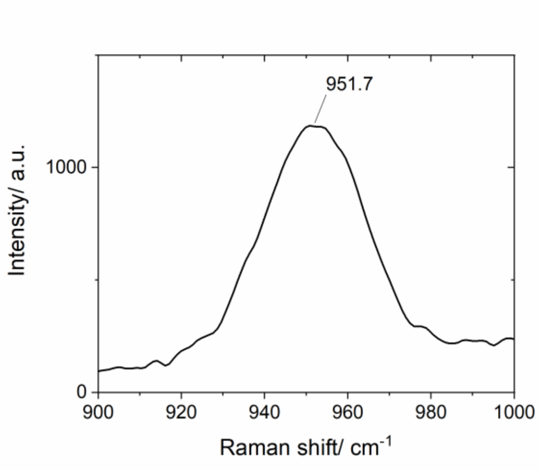

(b)

Figure S3. (a) Raman spectra of the precipitate formed in the urea–urease–calcium chloride reaction with broad PO_4 peaks at ~420, 586 and 952 cm^{-1} . The composition of the reactant solution was [urease] = 20 u/ml urease, [$H_2PO_4^-$] = 0.034 M, [urea] = 0.08 M, [HCl] = 3 mM, and [$CaCl_2$] = 4 mM. (b) Enlargement of peak at 952 cm^{-1} indicative of amorphous calcium phosphate (ACP).

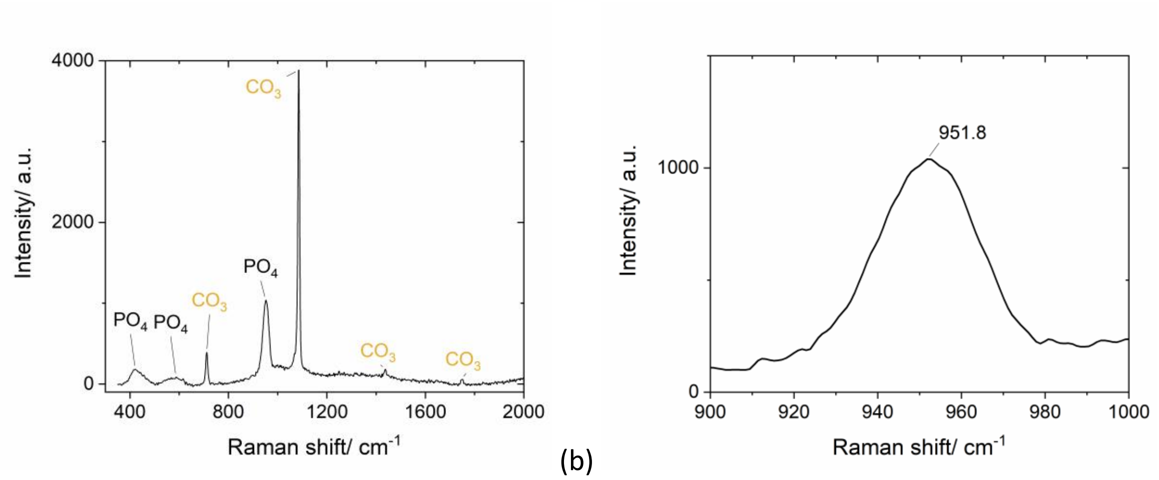

Figure S4. (a) Raman spectrum from precipitate taken during the oscillatory reaction with: [$CaCl_2$] = 0.25 M, [HCl] = 5 mM, [urease] = 30 u/ml, [$H_2PO_4^-$] = 0.05 M, [urea] = 0.5 M with broad PO_4 peaks at ~420, 586 and 952 cm^{-1} and CO_3 peaks at 712, 1086, 1437 and 1747 cm^{-1}. (b) Enlargement of peak at 952 cm^{-1} indicative of amorphous calcium phosphate (ACP).

(a)

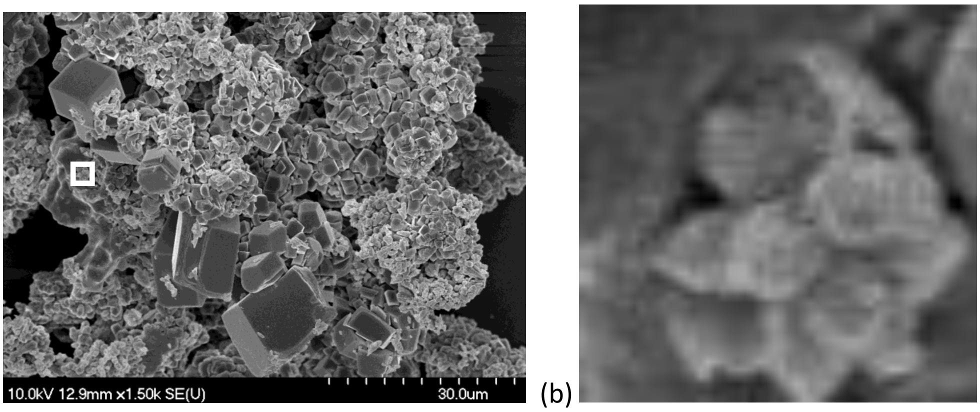

Figure S5. (a) SEM images of the dried precipitate formed in the reaction and (b) 3 µm x 3 µm section shown in (a). Concentrations were: [$CaCl_2$] = 0.25 M, [HCl] = 5 mM, [urease] = 30 u/ml, [$H_2PO_4^-$] = 0.05 M and [urea] = 0.5 M.

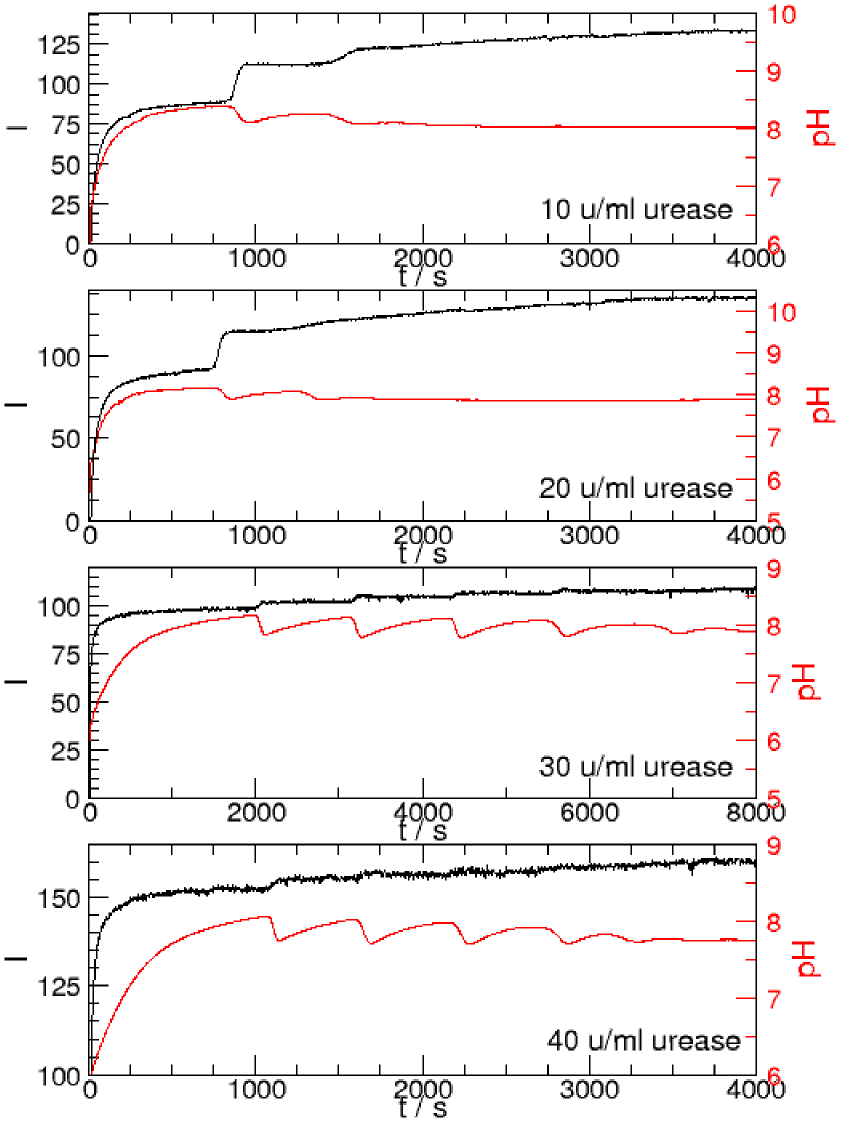

Figure S6. Greyscale intensity (I) of images and pH in time in reaction with concentrations: [$CaCl_2$] = 0.25 M, [urea] = 0.50 M, and [HCl] = 5 mM and (a) [urease] = 10 u/ml, [$H_2PO_4^-$] = 0.017 M (b), [urease] = 20 u/ml, [$H_2PO_4^-$] = 0.034 M (c), [urease] = 30 u/ml, [$H_2PO_4^-$] = 0.051 M (d), [urease] = 40 u/ml, [$H_2PO_4^-$] = 0.068 M.

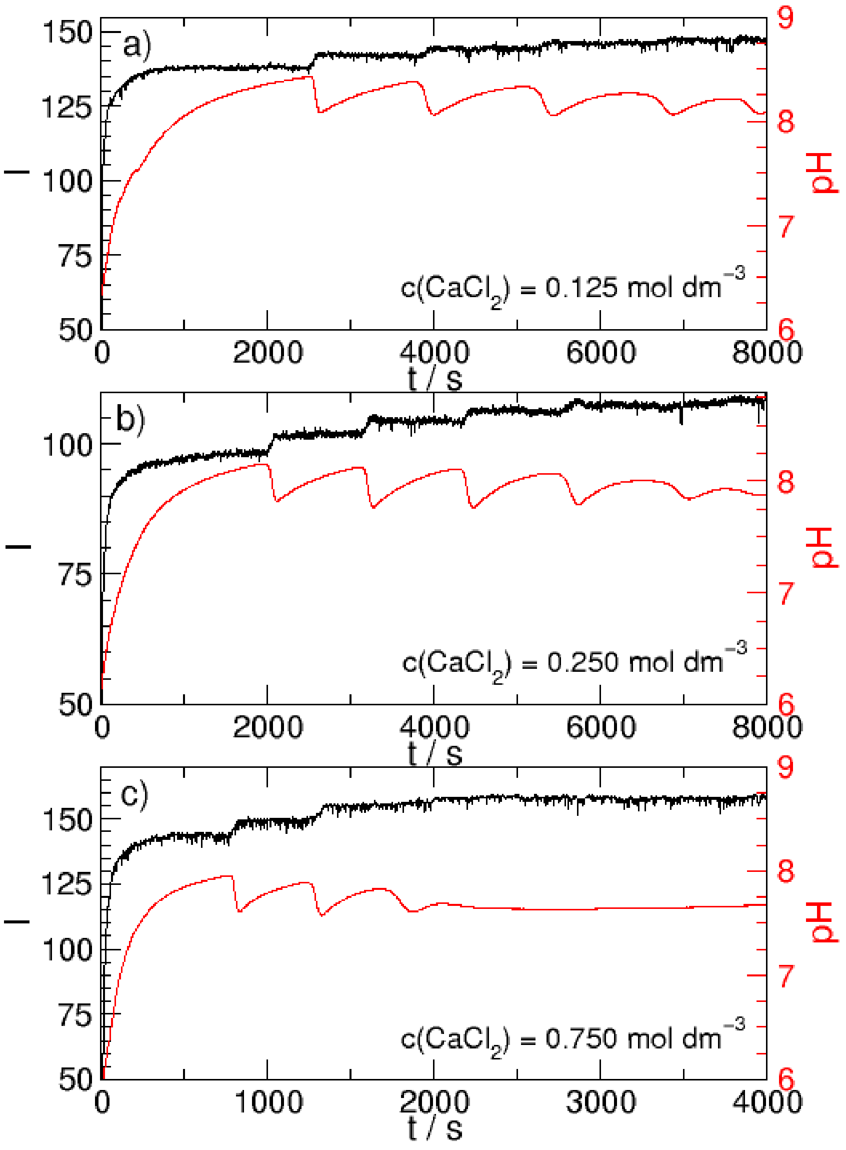

Figure S7. Greyscale intensity (I) of images and pH in time in reaction with concentrations [urea] = 0.50 M, [HCl] = 5 mM, [urease] = 30 u/ml, [$H_2PO_4^-$] = 0.05 M and [$CaCl_2$] = (a) 0.125 M, (b), 0.25 M (c), and (c) 0.75 M.

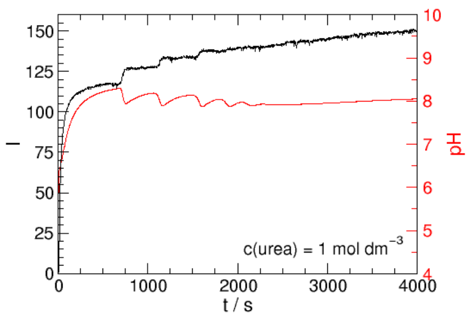

Figure S8. Greyscale intensity (I) of images and pH in time of reaction with [urea] = 1.0 M, [$CaCl_2$] = 0.25 M, [HCl] = 5 mM, [urease] =20 u/ml and [$H_2PO_4^-$] = 0.05 M.

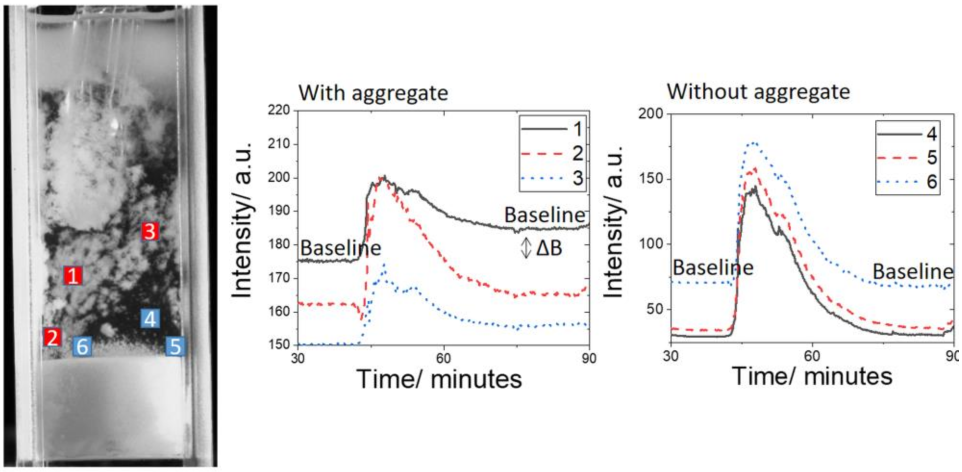

Figure S9. Periodic nucleation and aggregation of calcium phosphate in a cuvette (10 mm width) with low stirring rate (200 rpm) where [urea] = 0.5 M, [HCl] = 5 mM, $[CaCl_2]$ = 0.25 M, [urease] = 30 u/ml, $[H_2PO_4$-] = 0.05 M. Image of the cuvette where the white agglomerates of precipitate are deposited on the walls and average intensity from areas in the cuvette with aggregate (1 – 3) and without aggregate (4 – 6) where numbers 1 – 6 in the plots correspond to the areas indicated on the image. Baseline intensity and change in baseline intensity are also indicated.

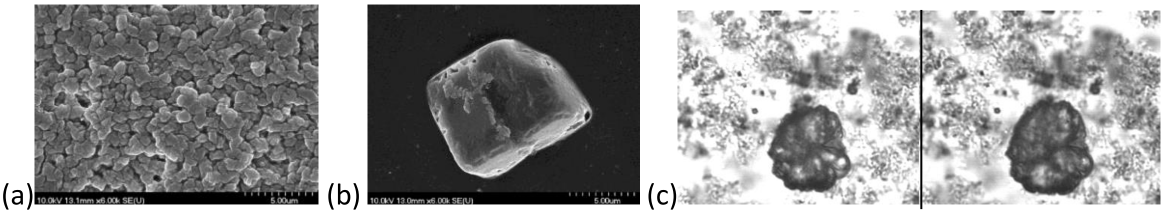

(a) (b) (c)

Figure S10. SEM images of the dried precipitate removed at (a) 80 s and at (b) 2500 s in the reaction with [urease] = 20 u/ml, $[H_2PO_4^-]$ = 0.034 M, $[CaCl_2]$ = 0.25 M, [urea] = 0.50 M, and [HCl] = 0.005 M. (c) Consecutive optical microscope images of sample of solution (90 μm x 70 μm) in time showing amorphous precipitate and growth of larger crystalline structure.

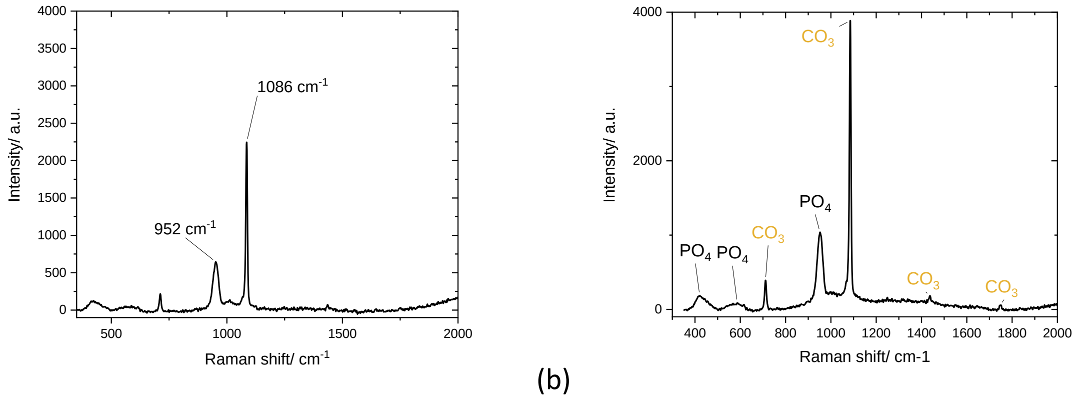

(a) (b)

Figure S11. Raman spectra of sample taken from (a) start of oscillatory period and (b) during oscillatory period, showing increase in calcite and calcium phosphate content. Concentrations: [urease] = 30 u/ml, $[H_2PO_4^-]$ = 0.05 M, $[CaCl_2]$ = 0.25 M, [urea] = 0.50 M, and [HCl] = 5 mM.

3. Model

The system involves enzyme urease in phosphate with substrate (urea), calcium chloride and acid under well-stirred, closed batch conditions. The main processes are (3.1) the enzyme catalysed reaction, (3.2) the equilibria that govern the pH and (3.3) the precipitation.

3.1 Enzyme-catalysed reaction

The enzyme catalysed hydrolysis of urea, U, yields ammonia and carbon dioxide:

(1) $CO(NH_2)_2 + H_2O \xrightarrow{urease} 2NH_3 + CO_2$

where the rate of the enzyme catalysed reaction is given by:

$$v = \frac{k_1 E_T U}{\left(K_M + U(1+\frac{U}{K_S})\right)\left(1+\frac{[NH_4^+]}{K_P}\right)\left(1+\frac{K_{es2}}{[H^+]}+\frac{[H^+]}{K_{es1}}\right)} \quad (E1)$$

and k_1 is the turnover number (s^{-1}), $[E]_T$ is the concentration of enzyme (M), K_M is the Michaelis constant, K_{es2} and K_{es1} are protonation equilibria of the substrate-enzyme complex. Substrate and product inhibition terms are included: K_u = equilibrium constant for uncompetitive substrate inhibition and K_p = equilibrium constant for non-competitive product inhibition.

3.2 Equilibria

The pH is determined by the following reversible reactions (for simplicity acid is included as H^+ rather than H_3O^+):

$NH_4^+ \rightleftharpoons NH_3 + H^+$ pK_a = 9.25 rate2 = $k_2[NH_4^+] - k_{2r}[NH_3][H^+]$ (E2)

$CO_2 + H_2O \rightleftharpoons H^+ + HCO_3^-$ pK_a = 6.35 rate3 = $k_3[CO_2] - k_{3r}[HCO_3^-][H^+]$ (E3)

$HCO_3^- \rightleftharpoons H^+ + CO_3^{2-}$ pK_a = 10.25 rate4 = $k_4[HCO_3^-] - k_{4r}[CO_3^{2-}][H^+]$ (E4)

$H_2O \rightleftharpoons H^+ + OH^-$ pK_w = 14 rate5 = $k_5 - k_{5r}[OH^-][H^+]$ (E5)

$H_3PO_4 \rightleftharpoons H_2PO_4^- + H^+$ pK_a = 2.12 rate6 = $k_6[H_3PO_4] - k_{6r}[H_2PO_4^-][H^+]$ (E6)

$H_2PO_4^- \rightleftharpoons HPO_4^{2-} + H^+$ pK_a = 7.21 rate7 = $k_7[H_2PO_4^-] - k_{7r}[HPO_4^{2-}][H^+]$ (E7)

$HPO_4^{2-} \rightleftharpoons PO_4^{3-} + H^+$ pK_a = 12.3 rate8 = $k_8[HPO_4^{2-}] - k_{8r}[PO_4^{3-}][H^+]$ (E8)

The desorption of gaseous CO_2 or NH_3 from the surrounding solution are not included here as the behaviour is qualitatively reproduced in sealed containers, nor H_2CO_3 as this species rapidly forms CO_2.

3.3 Coupling to precipitation

The calcium precipitates with both the inorganic carbon and phosphorous. The precipitation of calcium phosphate is complicated involving many crystalline species such as hydroxyapatite ($Ca_{10}(PO_4)_6(OH)_2$) and tricalcium phosphate ($Ca_3(PO_4)_2$). There are various types of amorphous calcium phosphate (ACP) that usually precipitate first in solutions, where some PO_4^{3-} ions may be replaced by HPO_4^{2-} and OH^- or even CO_3^{2-} or other ions giving different ratios of Ca/P. For simplicity, we include only amorphous tricalcium phosphate here:

$$3Ca^{2+} + 2PO_4^{3-} + nH_2O \rightarrow Ca_3(PO_4)_2.nH_2O\ (s) \qquad (E9)$$

For precipitation of calcium carbonate:

$$Ca^{2+} + CO_3^{2-} \rightarrow CaCO_3\ (s) \qquad (E10)$$

Calcium carbonate has various polymorphs; amorphous calcium carbonate (ACC), calcite, aragonite and vaterite. We have evidence of calcite formation at long times from the Raman spectra and SEM. The rate of formation of solid depends on the supersaturation, S, defined here as the ratio of the ion product to the solubility product K_{sp}:

$$S = IP/K_{sp} \qquad IP = \{Ca^{2+}\}^3\{PO_4^{3-}\}^2 \quad \text{or } \{Ca^{2+}\}\{CO_3^{2-}\} \qquad (E11)$$

where {} denotes activities: {} = $\gamma_i c_i$ where c_i is the concentration of the ith species and γ is the activity coefficient that depends on ionic strength: $I = 0.5\sum c_i z_i^2$. Precipitation involves nucleation, growth, aggregation/breakage and Ostwald ripening. We only consider nucleation here as the experiments suggest that nucleation occurs periodically. The steady state nucleation rate, J, in number of nuclei per unit volume per unit time, from classical nucleation theory is given by:

$$J = AS\exp(-B/(\ln S)^2) \qquad (E12)$$

Where A is the pre-exponential factor and $B = C_m\sigma^3\omega^2/(kT)^3$ where C_m is a shape factor (for spheres $C_m = 16\pi/3$), ω is the molecular volume, σ is the surface tension of the nucleating surface in water, k is the Boltzmann constant and T is the temperature. To imitate burst nucleation of spheres of precipitate of a critical radius, r_c then the rate of production of solid, in moles per unit volume of solution per unit time, was approximated by:

(1) $d[ATCP]/dt = p(S_1)K_{J1}S_1\exp(-B_1/(\ln S_1)^2)$

(2) $d[CaCO_3]/dt = p(S_2)K_{J2}S_2\exp(-B_2/(\ln S_2)^2)$

where $K_J = AV_c/V_m$ with V_m is the molar volume; $_1$ is for ATCP and $_2$ is for $CaCO_3$. The critical volume V_C depends on the radius: $r_c = 2\omega\sigma/(kT\ln S)$ and hence $K_J = K_J'/(\ln S)^3$ where $K_J' = 4\pi/3*(2\omega\sigma/kT)^3$. Inspired by the theory of LaMer, to delay the onset of nucleation the parameter $p(S)$ was introduced as a probability factor which depends on supersaturation:

$$S \geq S_{crit1} \qquad p = 1$$

The value of S_{crit1} was given by $S_{crit1} = 1 + R_1[Ca^{2+}]/x$ where x is the inorganic counterion and R_1 is a constant. The probability of nucleation depends on the ratio of metal ion, A, to counterion, B. A step function or a Gaussian probability function were used in earlier work with maximum probability at [A] = [B] and an decreasing probability of nucleation when A/B increased.[3] These simulations also took incorporated the stochastic nature of precipitation. Here, the dependence of S_{crit} on the ratio $[Ca^{2+}]/[PO_4^{3-}]$ was intended to take into account the fact that precipitation was less likely as the concentration of phosphate was decreased for experiments where $[Ca^{2+}] > [PO_4^{3-}]$. With $[Ca^{2+}] < [PO_4^{3-}]$, all available calcium was consumed as soon as precipitation commenced; this scenario was not explored in simulations.

The nucleation terminated when $S \leq S_{crit2} = 1 + R_2[Ca^{2+}]/x$ and then $p = 0$. This is different from the LaMer model, in which precipitation initiated and terminated above a single threshold. We found that oscillations occurred for $1 < S_{crit2} < S_{crit1}$; a better fit to experimental data was obtained using the same dependence of S_{crit2} on the ratio $[Ca^{2+}]/[PO_4^{3-}]$ as S_{crit1} but with $R_2 << R_1$.

2. Equations and parameters

There are 15 chemical species in reactions 1 – 6 (not including water). This results in 15 coupled odes. The values of all the rate constants and parameters taken in this work are shown in Table 1. The equilibria rate constants are well established[4], but the enzyme constants vary depending on the source. The enzyme constants are in line with earlier work[5]. The enzyme in the ode file is given in units/ml to more easily compare to experiments and the maximum rate for E_T = 19 unit/ml is v_{max} = E_T*k_{1e} = 19 units/ml*2 x 10^{-5} M/units/ml/s = 3.8 x 10^{-4} M/s (and k_1 in s^{-1} = k_{1E} /5.3 x 10^{-8} M/units/ml).

The ionic strength here ranged from I = 0.05 – 2 M and can be assumed to be constant as it was dominated by large initial concentrations of ions from $CaCl_2$. The activity coefficients can be calculated from the Pitzer equation for high ionic strength, however the latter includes empirical parameters that were not evaluated in the complex mixtures used here.[6] It is expected that the inclusion of activity coefficients will not impact the qualitative results and so we used concentrations in the simulations, rather than activities. The aim of the simulations was to help explain the dynamic behaviour, rather than reproduce the results quantitatively.

The K_{sp} of calcite was used for $CaCO_3$ precipitation. For ACP we may have a combination of species, and/or incorporation of additional ions and the solubility product K_{sp} for amorphous compounds cannot be precisely determined but a range of estimates have been reported, from 3 x 10^{-17} to 2 x 10^{-33}, depending on the pH and other factors.[7] For simplicity, we included only ATCP in the model and took the value of K_{sp} = 3 x 10^{-17}. We estimated values for K_J' and B using available data for $CaCO_3$; data for ATCP was more difficult to obtain and hence we used reasonable values based on comparison with $CaCO_3$. The values of R_1 and R_2 were taken to best match the experimental results. The pre-exponential factor A can be approximated by various expressions but is usually experimentally obtained with a wide variety of values from 10^{12} – 10^{25} cm^{-3} s^{-1}; we took A = 10^{12} cm^{-3} s^{-1}; ω = 6 x 10^{-23} cm^3; σ = 70 J/ cm^2, and V_m = 3.7 x 10^{-2} dm^3/mol for $CaCO_3$, of molecular mass 100.1 g/mol. The value of A is larger for amorphous compounds, and the surface energy reduced, hence a larger value of K_J' was used for ATCP compared to $CaCO_3$ and the value of B was lower. We emphasise that the goal here was to provide insight to the temporal behaviour. The rate equations

were solved using XPPaut[8] with integration method “cvode”. The ode file for the model is included in the Appendix.

Table 1. Rate constants (25 °C) and precipitation parameters.

Equilibria rate constants[4b, 8]	k_2 s^{-1}	k_{2r} M^{-1} s^{-1}	k_3 s^{-1}	k_{3r} M^{-1} s^{-1}	k_4 s^{-1}	k_{4r} M^{-1} s^{-1}	k_5 M s^{-1}	k_{5r} M^{-1} s^{-1}
	24	4.3×10^{10}	0.037	7.9×10^{4}	2.8	5×10^{10}	1×10^{-3}	1×10^{11}
	k_6 s^{-1}	k_{6r} M^{-1} s^{-1}	k_7 s^{-1}	k_{7r} M^{-1} s^{-1}	k_8 s^{-1}	k_{8r} M^{-1} s^{-1}		
	$7.6x10^{8}$	$1x10^{11}$	$6.2x10^{2}$	$1x10^{10}$	0.05	$1x10^{11}$		
Enzyme constants[5]	k_1 s^{-1}	K_M M	K_{es1}	K_{es2}	K_U	K_P		
	377	3×10^{-3}	5×10^{-6}	2×10^{-9}	3	0.002		
Precipitation parameters<br>1 = $Ca_3(PO_4)_2$<br>2 = $CaCO_3$	K_{J1}' M s^{-1}	B_1	K_{J2}' M s^{-1}	B_2	R_1	K_{sp1}	K_{sp2}	R_2
	0.01	10	$1 x 10^{-6}$	300	$1 x 10^{-3}$	$3x 10^{-17}$	$3 x 10^{-9}$	$2 x 10^{-5}$

References

[1] aT. Tsuji, K. Onuma, A. Yamamoto, M. Iijima, K. Shiba, *Proceedings of the National Academy of Sciences of the United States of America* **2008**, *105*, 16866-16870; bC. Combes, C. Rey, *Acta Biomaterialia* **2010**, *6*, 3362-3378; cG. R. Sauer, W. B. Zunic, J. R. Durig, R. E. Wuthier, *Calcified Tissue International* **1994**, *54*, 414-420.

[2] G. Penel, C. Delfosse, M. Descamps, G. Leroy, *Bone* **2005**, *36*, 893-901.

[3] H. K. Henisch, *J. Cryst. Growth* **1986**, *76*, 279-289.

[4] aM. Eigen, *Angew. Chem.-Int. Edit.* **1964**, *3*, 1; bX. G. Wang, W. Conway, R. Burns, N. McCann, M. Maeder, *J. Phys. Chem. A*, *114*, 1734-1740.

[5] aB. Krajewska, *Journal of Molecular Catalysis B-Enzymatic* **2009**, *59*, 9-21; bB. Krajewska, S. Ciurli, *Plant Physiol. Biochem.* **2005**, *43*, 651-658.

[6] F. Pérez-Villaseñor, G. A. Iglesias-Silva, K. R. Hall, *Ind. Eng. Chem. Res.* **2002**, *41*, 1031-1037.

[7] W. J. E. M. Habraken, J. Tao, L. J. Brylka, H. Friedrich, L. Bertinetti, A. S. Schenk, A. Verch, V. Dmitrovic, P. H. H. Bomans, P. M. Frederik, J. Laven, P. Van Der Schoot, B. Aichmayer, G. De With, J. J. DeYoreo, N. A. J. M. Sommerdijk, *Nature Communications* **2013**, *4*.

[8] P. L. Brezonik, *Chemical kinetics and process dynamics in aquatic systems*, CRC Press, **1994**.

[9] Ermentrout, G. B. Simulating, Analyzing, and Animating Dynamical Systems: A Guide to XPPAUT for Researchers and Students; SIAM: Philadelphia, **2002**.

Appendix 1: Ode file for XPPaut

```
#urea-urease precipitation reaction: rate equations

# rate of enzyme reaction
o(E,U,H)=k1e*E*U/((1+KE2/H+H/KE1)*(KME+U*(1+U/Ku))*(1+NH4/Kp))

# rate of nucleation
f(Ca,PO4)=P1*Kj1*(1/(ln(Ca^3*PO4^2/Ksp1))^3)*(Ca^3*PO4^2/Ksp1)^n*exp(-
B1/(ln(Ca^3*PO4^2/Ksp1))^2)
g(Ca,CO3)=P2*Kj2*(1/(ln(Ca*CO3/Ksp2))^3)*(Ca*CO3/Ksp2)^n*exp(-B2/(ln(Ca*CO3/Ksp2))^2)

#kinetic equations
U'=-o(E,U,H)
NH3'=2*o(E,U,H)+k2*NH4-k2r*NH3*H
NH4'=-k2*NH4+k2r*NH3*H
CO2'=o(E,U,H)-k3*CO2+k3r*H*HCO3
HCO3'=k3*CO2-k3r*HCO3*H-k4*HCO3+k4r*CO3*H
CO3'=k4*HCO3-k4r*CO3*H-g(Ca,CO3)
H3PO4'=k6r*H*H2PO4-k6*H3PO4
H2PO4'=k7r*H*HPO4-k7*H2PO4-k6r*H*H2PO4+k6*H3PO4
HPO4'=-k7r*H*HPO4+k7*H2PO4-k8*HPO4+k8r*H*PO4
PO4'=k8*HPO4-k8r*H*PO4-2*f(Ca,PO4)
H'=k2*NH4-k2r*NH3*H+k4*HCO3-k4r*CO3*H+k5-k5r*H*OH+k3*CO2-k3r*HCO3*H-
k7r*H*HPO4+k7*H2PO4+k8*HPO4-k8r*H*PO4-k6r*H*H2PO4+k6*H3PO4
OH'=k5-k5r*H*OH
Ca'=-g(Ca,CO3)-3*f(Ca,PO4)
CaCO3'=g(Ca,CO3)
CaPO4'=f(Ca,PO4)

#Precipitation
Scrit1p=1+R1*Ca/PO4
Scrit2p=1+R2*Ca/PO4
Scrit1c=1+R1*Ca/CO3
Scrit2c=1+R2*Ca/CO3

global 1 {Ca^3*PO4^2/Ksp1-Scrit1p} {P1=1}
global -1 {Ca^3*PO4^2/Ksp1-Scrit2p} {P1=0}
global 1 {Ca*CO3/Ksp2-Scrit1c} {P2=1}
global -1 {Ca*CO3/Ksp2-Scrit2c} {P2=0}

aux pH=-log(H)/log(10)
aux Sc=Ca*CO3/Ksp2
aux Sp=Ca^3*PO4^2/Ksp1

#parameters
par kJ2=1e-6,B2=300,kJ1=0.01,B1=10,R1=1e-3,R2=1e-5,n=1
par Ksp2=3e-9,Ksp1=3e-17,P1=0,P2=0
par k1e=3e-5,E=30,KME=0.003,Ku=3,KP=0.002,KE1=5e-6,KE2=2e-9
```

```
par k2r=4.3e10, k2=24, k3r=7.9e4, k3=0.037,k4r=5e10, k4=2.8, k5r=1e11, k5=1e-3
par k6r=1e11,k6=7.6e8, k7r=1e10,k7=6.2e2,k8r=1e11,k8=0.05

#initial conditions
init U=0.5, H=3e-3, OH=1e-11, Ca=0.25,H2PO4=5e-2

#numerical stuff
@ total=8000,dt=0.1,tol=1e-13, atol=1e-9, meth=cvode
@ xplot=t,yplot=pH,xhi=10000,ylo=6,yhi=9
@ maxstor=10000000
@ bound=1e20
done
```
